# Response and Plasticity of a Cleaning Mutualism Following Short Term Reductions in Habitat Availability

**DOI:** 10.1002/ece3.73348

**Published:** 2026-04-16

**Authors:** R. L. Gunn, C. G. Obst, P. Vetter

**Affiliations:** ^1^ Animal Evolutionary Ecology, Institute of Evolution and Ecology University of Tübingen Tübingen Germany

**Keywords:** behavioural plasticity, behavioural responses, cleaning mutualism, coral reefs, environmental change, habitat manipulation, positive species interactions

## Abstract

Species interactions, facilitated by animal behaviour, drive global biodiversity, yet species interactions are being disrupted by environmental change across ecosystems. On coral reef ecosystems, cleaning mutualisms where a ‘cleaner’ species removes parasites from a ‘client’ species at dedicated cleaning ‘stations’ on live coral are directly impacted by ongoing declines in live coral due to environmental change. Using cleaning mutualisms as a model system, we hypothesised that reduced habitat availability will result in a decline in mutualism‐associated behavioural expression. Through an in situ manipulation experiment on a Caribbean coral reef, we placed nets over obligate cleaner goby (*Elacatinus lobeli*) cleaning stations, reducing live coral and refuge access. We identified consistent declines in the quality (cleaning rate) and quantity (cleaning duration) of cleaning interactions, cleaner selectivity for specific clients and client signalling (posing) following habitat manipulations and an increase in cleaner responsiveness (flight initiation distance), whilst the abundance and diversity of the local reef fish community remained relatively unchanged. We also found evidence of behavioural plasticity, with mutualism‐associated behaviours returning to pre‐disturbance levels after the manipulation was removed. Whilst the functioning of marine cleaning mutualisms declines when habitat availability is reduced, plasticity in mutualism‐associated behaviours can maintain cleaner–client interactions. Identifying thresholds of change beyond which such plasticity is no longer viable, resulting in a mutualism breakdown, is an important next step in preserving one of the world's most threatened ecosystems.

## Graphical abstract

On coral reef ecosystems, cleaning mutualisms where a ‘cleaner’ species removes parasites from a ‘client’ species at dedicated cleaning ‘stations’ on live coral are directly impacted by ongoing declines in live coral due to environmental change. We reduced cleaner fish access to live habitat and identified declines in mutualist service quality and quantity, as well as recovery following the removal of habitat manipulations. Whilst the functioning of marine cleaning mutualisms declines when habitat availability is reduced, marine cleaning mutualisms may could show a level of resilience via behavioural plasticity in response to short term changes in environmental conditions.

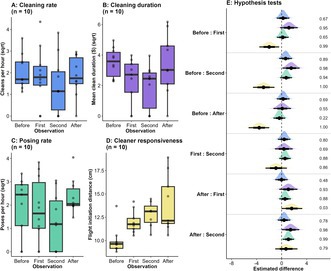

## 1 Introduction

Species interactions played a fundamental role in the organisation and diversification of life on Earth (Thompson 1999). Every species contributes to a complex network of ecological interactions, which are facilitated by animal behaviour. Ecological outcomes arise from behavioural processes and scale up to impact species interactions (Werner 1992; Keith et al. 2023). Positive, mutualistic interactions, whereby two species cooperate beneficially, are present in all ecosystems, from plant–pollinator mutualisms driving global food webs (Klein et al. 2007), to coral–zooxanthellae symbioses on coral reefs providing the foundational structure and habitat for one of the world's most productive ecosystems (Plaisance et al. 2011). Mutualisms often arise from behavioural interactions (Gordon 2011). For example, on coral reefs, dedicated ‘cleaner’ species remove and consume parasites from ‘client’ species within a service‐resource mutualism (Leung and Poulin 2008). This mutualism is dependent on complex behavioural expression via signalling from both cleaners and clients (Caves 2021). Almost every organism is involved in a mutualistic interaction, with these interactions giving rise to biodiversity via adaptation and co‐evolution (Bronstein 2015). Mutualistic interactions therefore offer a novel tool to quantify aspects of ecosystem function across multiple ecological levels.

The outcome (positive–negative) of a biotic interaction lies on a continuum and is determined by the balance of cost: benefit ratios (Gómez et al. 2023). Mutualisms are the most variable species interaction compared to competition and predation (Chamberlain et al. 2014) and mutualistic cost: benefit ratios are dependent on local conditions (Gunn and Michiels 2025). Changes in these conditions can shift alliances between interacting parties, which in turn may result in mutualistic interactions breaking down (Kiers et al. 2010). In the African Savannah, the removal of herbivores affects ant‐plant mutualisms and results in a switch from a positive, mutualistic interaction to a negative, antagonistic interaction (Palmer et al. 2008). In arctic ecosystems, plant–pollinator networks are becoming less robust due to glacial retreat (Conti et al. 2024). On coral reefs, rising temperatures result in the breakdown of the coral–zooxanthellae mutualism, which is the direct cause of coral bleaching (Helgoe et al. 2024). In fact, biotic mechanisms, specifically altered species interactions, are a more important mechanism behind climate‐change related threats to populations than the direct effects of rising temperatures, i.e., species interactions can drive responses to environmental change (Ockendon et al. 2014; Åkesson et al. 2021).

Environmental changes are ubiquitous across all ecosystems (Watson et al. 2018), but coral reefs are the first ecosystem to have surpassed a climate ‘tipping point’ (Lenton et al. 2025). In the Caribbean, stony coral tissue loss disease (SCTLD) is inducing high rates of coral mortality (Alvarez‐Filip et al. 2011), with negative implications for reef fish communities (Swaminathan et al. 2024). Furthermore, the largest, most widespread mass coral bleaching event occurred between 1st January 2023 and 30th September 2025, impacting around 84.4% of the world's coral (NOAA Coral Reef Watch 2024). Both SCTLD and bleaching events are exacerbating declines in structural complexity, the foundational building block for coral reef ecosystem structure and function (Graham and Nash 2013). Coral reef inhabitants depend on healthy, live coral for refuges (Boström‐Einarsson et al. 2018), nutrients (Gunn et al. 2022) and mutualistic cleaning services (Côté and Brandl 2021). Cleaning stations of obligate cleaner fishes in both the Caribbean (*Elacatinus* spp) and the coral triangle (*Labroides* spp.) display distinct habitat characteristics (Côté and Brandl 2021; Whittey et al. 2021) and microhabitat quality can be associated with increased cleaning service quality (Whittey et al. 2021). Furthermore, the maintenance of *Elacatinus* cleaning mutualisms is more dependent on local, rather than wider community biodiversity (Dunkley et al. 2020). Local reef fish communities are also more likely to be directly affected by local‐scale environmental changes.

There is some evidence that marine cleaning mutualisms are being disrupted by environmental change. In response to the 2016 El Niño event and cyclone activity, the density of the obligate cleaner *Labroides dimidiatus* declined by 80% on the Great Barrier Reef, and individuals became less strategically sophisticated and selective for clients that would allow the cleaners to maximise nutritional resources (Triki et al. 2018). Similarly, the coral heads utilised by *Elacatinus* cleaner gobies are among those impacted by SCTLD, and *Elacatinus* abundance on these coral heads was 50% lower in areas that had SCTLD outbreaks compared to non‐affected sites (Budd et al. 2024). Nonetheless, *Elacatinus* density remained consistent overall, indicating a switch in habitat away from affected coral (Budd et al. 2024). *Elacatinus* cleaners may therefore show a degree of plasticity in habitat selection and perhaps in mutualism‐associated behaviours in response to changing conditions.

Behaviour is often the first tool used by organisms to respond to changing conditions (Gunn et al. 2021). Behavioural plasticity allows individuals to display rapid changes in behavioural traits in response to changes in the environment (Komers 1997). However, the immediate, short‐term responses of positive species interactions to environmental changes, and the potential for interacting parties to display plasticity in responses, remain relatively unknown (Lewis and Altieri 2025). There is an inherent link between behaviour and species interactions (Werner 1992). Understanding how changing environmental conditions affect the short‐term functioning of mutualism‐associated behaviours is therefore the foundation for predicting the future persistence of functionally important mutualisms. As studies on animal behaviour are sensitive to the study environment (Calisi and Bentley 2009), there needs to be a focus on field‐based, in situ research, yet laboratory studies tend to dominate (Gunn et al. 2021). A more balanced representation of field‐based studies would provide valuable insight into how context‐dependent species interactions respond to changing conditions.

We aim to determine how mutualistic interactions respond to short‐term changing conditions, using the *Elacatinus* cleaning mutualism on a Caribbean coral reef as a model system. We reduce cleaner access to live coral on cleaning stations for a period of 11 days in an in situ habitat manipulation and observe cleaning stations before, during and after manipulations in order to (1) quantify if and how reduced refuge access and habitat availability affects cleaner–client interaction and (2) quantify any evidence of plasticity in responses after the removal of manipulations that could allow a functionally important service–resource mutualism to be maintained through environmental variability. We measure multiple aspects of the *Elacatinus* cleaning mutualism, from individual cleaner behaviours to local scale reef fish biodiversity, as altered species interactions can scale up to have impacts on communities at higher ecological levels.

## 2 Methods

### 2.1 Study Site and Species

Between 20th June–12th July 2024, 20 *Elacatinus lobeli* stations were selected along a 250 m stretch of fringing reef around the south side of the island of Utila, Honduras (16^o^ 05′ 17.96′′N 86^o^ 54′ 38.27′′W). Data collection occurred approximately 18 months into a mass coral bleaching event (NOAA Coral Reef Watch 2024). The bleaching event caused widespread bleaching around Utila by late 2023 (Green et al. 2026), and there was some evidence of bleaching resulting in coral death at our study site in comparison to 2023 (R. Gunn, unpubl.). Nonetheless, we ensured that all focal cleaning stations did not show signs of active bleaching at the time of data collection.

All data were collected via snorkel between 0.5 and 5 m depth. Focal cleaning stations were at least 3 m away from any other active cleaning station. The depth of each cleaning station was recorded, as well as the number of cleaners on each station. All focal stations were occupied by the Caribbean Neon Goby, *Elacatinus lobeli*. *E. lobeli* are small (~4 cm) obligate cleaner fish that occupy cleaning stations on boulder/other non‐branching coral heads (Whiteman and Côté 2002).

### 2.2 Baseline Observations

We conducted preliminary observations at the 20 focal cleaning stations in order to establish a ‘baseline’ to which the experimental manipulations could be compared.

#### 2.2.1 Behavioural Observations

A GoPro Hero 10 camera was placed in the vicinity of each focal cleaning station and left until the battery was depleted (between 40 and 104 min). Videos were analysed in BORIS (Friard and Gamba 2016), with behavioural quantification following Gunn and Michiels (2025). For each station, we recorded the duration and rate (cleans per hour) of all cleans, as well as the rate of poses made by clients. Posing is a well‐defined signalling behaviour, whereby potential clients hover at a cleaning station and display fin flaring and/or gill opening (Côté et al. 1998). We recorded the number of cleaners at focal stations that engaged in each cleaning interaction. Where possible, we distinguished between cleaners at a cleaning station based on size, as one individual was often clearly larger. Where this was not the case, we used the positioning of cleaners on the station, as different individuals tended to occupy specific points on the cleaning station. We were therefore able to quantify all cleaning interactions that occurred across all the focal stations within the observation period.

#### 2.2.2 Cleaner Flight Initiation Distance (FID)

We quantified cleaner ‘responsiveness’, quantified as FID, with longer FID values indicating a more responsive cleaner (Gunn and Michiels 2025; Wilson et al. 2014). We calculated a cleaner FID using a simple disturbance test (Dunkley et al. 2019; Gunn and Michiels 2025). We approached each cleaning station with a 7 cm long polystyrene oval ball at the end of a 50 cm bamboo stick with a cm measurement tape attached along the bamboo stick. A GoPro Hero 10 was attached to the end of the bamboo stick, with a top–down perspective over the polystyrene ball. The stick, held by a snorkeler at arm's length, was moved just over the surface of the cleaning station toward the resident cleaners. We used ImageJ to quantify cleaner FIDs. Where more than one cleaner could be measured at a single cleaning station, we distinguished between individuals based on size.

#### 2.2.3 Client Community Metrics

We quantified multiple aspects of the local reef fish community. We extracted one frame per minute from the video footage used to quantify cleaner–client interactions at each focal cleaning station. Images were analyzed chronologically, recording the identity and abundance of all species within the videos. This data was then used to calculate actual and ‘potential’ client species richness, as every species recorded was within the vicinity of a focal cleaning station. As there were a different number of screenshots for each video due to differences in recording length, we calculated total potential client abundance as a rate, quantifying the number of fish at a focal cleaning station per hour.

We used space use and any distinct markings to distinguish returning individuals from new individuals within a species. By watching the cleaning videos, analysing the screenshots chronologically and having the same researcher complete all data extraction, we minimised pseudoreplication whereby the same individual fish could be recorded multiple times.

#### 2.2.4 Cleaner Selectivity

To determine if cleaners show preferences for specific client species/families, we calculated a cleaner selectivity index (Gunn and Michiels 2025), based on the Vanderploeg–Scavia Index (Vanderploeg and Scavia 1979) for zooplankton diet preferences, which has more recently been modified to quantify reef fish diet preferences (Zambre and Arthur 2018). The cleaner selectivity index calculates the extent to which a cleaner provides a service to a specific client family at any given station, given the availability of these families, such that

(1) $$W_{i}=\frac{\left(\frac{r_{i}}{p_{i}}\right)}{\sum_{n=0}^{n=i}\left(\frac{r_{i}}{p_{i}}\right)}$$

whereby *r* is the proportion of total time spent cleaning each client species per station, and *p* is the availability of each client species (Gunn and Michiels 2025, Equation S1). Selectivity *E* was then calculated as

(2) $$E=\frac{\left[W_{i}-\left(\frac{1}{n}\right)\right]}{\left[W_{i}+\left(\frac{1}{n}\right)\right]}$$

whereby *n* is the species richness of clients cleaned at each station. The index produces values between −1: strong selectivity *against* a particular client family and 1: strong selectivity *for* a particular client family, with 0 indicating no preference (Gunn and Michiels 2025).

#### 2.2.5 Cleaning Station Size and Structure

We quantified the height, surface area and structural complexity of all focal cleaning stations. We filmed all cleaning stations as outlined in Whittey et al. (2021), extracted frames from the video footage and used these images to produce 3D models. We rendered models in Agisoft Metashape and analysed models using Rhinoceros 3D. Within the Rhinoceros 3D software, we calculated linear rugosity using a ‘virtual chain’ method with virtual 2 cm link length chains from Whittey et al. (2021). We then calculated the ratio between the length of the chain over the cleaning station and the flat length of the chain (Young et al. 2017), resulting in a rugosity value between 0, a highly rugose habitat, and 1, a completely flat plane.

### 2.3 Habitat Manipulation Set Up

All stations were randomly assigned to one of 3 treatment groups: Experimental (*n* = 10), Experimental control (*n* = 5) or Control (*n* = 5). We placed a 1 m^2^, 500 μm mesh, slightly weighted net over each experimental cleaning station. The mesh size prevented any disruption to coral/zooxanthellae feeding. We used the location of the cleaners on each cleaning station directly before net placement as the centre point for placing the net, ensuring the cleaners had first moved from this location and were not trapped under the net. For experimental control stations, nets were cut completely open, so live habitat access was not affected. Control stations had no manipulations. The control treatment therefore allowed us to quantify natural variation in cleaner–client interactions over time, whilst the experimental control treatment allowed us to quantify variation due to the mere presence of the manipulation.

Preliminary observations outlined above acted as a baseline prior to habitat manipulations (‘Before’). Nets remained on the station for 11 days, during which all 20 focal stations were observed twice: once within the first 6 days following manipulation (‘First’), and once in the final 5 days (‘Second’). Nets were then removed, and all stations were surveyed for a final time, between 2 and 8 days after net removal (‘After’). Each cleaning station therefore had four replicate surveys: Before, First, Second and After. Although the time‐frame of the study is short, resulting in a mismatch between real‐world disturbance time scales and the disturbance time‐scale of the study, behavioural responses can occur immediately in response to changing conditions, especially where changes directly impact the animal in question (Rahman and Candolin 2022). The duration of the habitat manipulation is appropriate to the aims and objectives of the study. Each replicate survey quantified eight variables of interest: cleaning rate, posing rate, cleaning duration, cleaner responsiveness, cleaner selectivity, potential client abundance, and richness and actual client species richness.

Cleaning stations are highly context‐dependent (Chamberlain et al. 2014), and as such, likely display natural variation even over short periods of time. Comparing absolute values for response variables between control and experimental values at each replicate could make ecological interpretation difficult as a result. It is therefore necessary to determine how experimental cleaning stations differ across the replicates, specifically in comparison to naturally expected variation over time, i.e., variation across replicates at control stations. For each variable across each treatment, we calculated the absolute difference between the three replicates following the habitat manipulation (‘First’, ‘Second’, ‘After’) and the baseline ‘Before’. For example, to quantify the difference between a First replicate and a Before replicate value:

(3) Absolute difference=First replicate value−Before replicate value

We repeated this for the Second and After replicates for each station. For the control treatment, we then calculated the mean relative difference across all control stations for each of the three replicates following the habitat manipulation.

To determine if the experimental control and the experimental treatments showed variation away from this expected variation over time, we then calculated the difference between the absolute difference for each replicate within each treatment (experimental control and experimental) and the equivalent mean relative difference calculated for the control treatment. For example, to quantify the relative difference between experimental first replicate values and control first replicate values:

(4) Absolute difference relative to control=First replicate experimental value−Mean first replicate control value

### 2.4 Statistical Analysis

All statistical analyses were conducted using R 4.2.1 (R core team 2019), using Bayesian hierarchical mixed effect models computed with the ‘brms’ package (Bürkner 2017) implemented in STAN (Stan Development Team 2022). Bayesian statistics are appropriate for small sample sizes and are now often used to analyse ecological and behavioural data (Gunn et al. 2023; Sheppard et al. 2024), including cleaning mutualism data (Gunn and Michiels 2025). In the Bayesian approach used here, posterior probabilities (PP) determine the probability with which a given hypothesis is supported. For example, a PP of 0.95 indicates there is a 95% probability that the tested hypothesis is supported. Evidence ratios (ERs) determine the extent of support for alternative hypotheses. ERs can be considered transformed posterior probability values such that $\mathrm{ER}=\frac{\mathrm{PP}}{\left(1-\mathrm{PP}\right)}$. Finally, credible intervals (CIs) are uncertainty intervals derived from the posterior distribution of model parameters. For example, a 95% CI indicates there is a 95% probability that the true value of a parameter falls within that range, given both the model and the data, and is calculated based on the 2.5% and 97.5% percentiles (Bürkner 2017). Bayesian approaches therefore provide a more nuanced understanding of variation in the data than the use of *p*‐values.

We elected to incorporate ‘Time’, in our case ‘Observation’ (Before, First, Second After), as a categorical rather than a discrete variable for statistical analyses. Logistically, it was not possible to observe every station every day or every station on a consistent timeframe. For example, water conditions meant that some focal stations could only be accessed on certain days. Treating time as a discrete variable as ‘days since disturbance’ would result in gaps due to missing days, making the ecological interpretation of changes over time more difficult.

We produced distribution plots to identify suitable distributions and square root transformed variables where necessary to improve model fits. All models were computed over four chains, with 15,000 iterations, a warmup of 5000 iterations and using weakly informative normal priors.

Cleaning station surface area and cleaning station ID were included as random effects in all models. Posterior mean Intraclass Correlation Coefficients (ICCs) were computed to quantify the model variability explained by each random effect (Nakagawa et al. 2017). We used a rough guideline of ICC values < 0.05 indicating ‘minimal’ group‐level clustering, 0.05–0.20 indicating ‘meaningful’ group‐level clustering and > 0.20 indicating ‘strong’ group‐level clustering (Nakagawa et al. 2017).

Trace plots, graphical posterior predictive checks, bulk effective sample sizes (ESS) and a convergence diagnostic (R‐hat, Vehtari et al. 2021) were used to assess model convergence and fit. Influential data points were identified using Pareto‐smoothed importance‐sampling leave‐one‐out cross‐validation (PSIS_LOO). Datapoints with values > 1 were assumed to have failed and were removed from the data, and points with values > 0.7 were considered to be influential. Influential data points were removed, models rerun and then readded when model outputs remained unchanged.

Slope estimates, 75% and 95% CI for posterior predictive distributions, were extracted from all models. One‐way hypothesis testing of a priori hypotheses was used to test for the strength of effects. We extracted posterior probabilities and ER for all hypothesis tests.

#### 2.4.1 Cleaning Station Size and Structure

As stations were randomly assigned to the treatment groups, we ran a multivariate Bayesian fixed effect model to determine if both cleaning station surface area and cleaning station rugosity varied between treatments. To improve model fits and to directly compare the experimental against both control groups, we merged the control and experimental control stations for this model. Treatment was then set as the explanatory variable.

For the experimental stations (*n* = 10), we ran a simple Bayes factor test to identify any negative, nonzero association between cleaning station rugosity and cleaning station surface area compared to a null model. In general, a Bayes factor of 1–3 indicates anecdotal evidence for a correlation, 3–10 indicates moderate evidence and > 10 indicates strong evidence for a correlation (Kass and Raftery 1995).

#### 2.4.2 Habitat Manipulation: Experimental Treatment

For the experimental cleaning stations (*n* = 10), we computed Bayesian mixed effect models to determine how each of the eight response variables varied across the habitat manipulation. ‘Observation’ (Before, First, Second, After) was set as the explanatory variable. For each model, we conducted nonlinear hypothesis tests to quantify the extent to which each response variable changed during the habitat manipulation.

#### 2.4.3 Habitat Manipulation: Differences Relative to Controls

For both Experimental and Experimental control stations, we computed additional models for all response variables, with relative difference for each behaviour compared to control stations as the response variable and Observation as the explanatory variable. For each model, we then conducted nonlinear hypothesis tests to determine how the relative change of response variables compared to controls differed at experimental stations/experimental control stations compared to control stations for each replicate survey (First, Second, After).

## 3 Results

### 3.1 General Summary

We conducted a total of 80 video behavioural observations across 20 focal cleaning stations, quantifying 428 cleans and 448 incidences of posing from ~66 h of footage. The number of cleaners per station ranged from 1–8, but only 4 interactions involved more than 2 cleaners. Cleaners serviced 26 different client species from across 16 different client families. We conducted 78 cleaner responsiveness tests during the duration of the study: one test per replicate observation (Before, First, Second, After) per station. In two cases, it was not possible to quantify cleaner responsiveness due to poor‐quality footage.

The surface area of focal cleaning stations ranged from 0.86 to 58.63 m^2^, and cleaning station rugosity ranged from 0.05 to 0.41. There was little evidence that cleaning station rugosity (Estimate: −1.1 (−4.40, 2.20), PP: 0.72, ER: 2.52) or surface area (Estimate: 0.0 (−0.07, 0.08), PP: 0.45, ER: 0.82) varied between control (*n* = 10) and experimental cleaning stations (*n* = 10). There was ‘moderate evidence’ for a negative correlation between cleaning station rugosity and cleaning station surface area (Bayes Factor: 3.93, PP: 0.95, Figure S1).

### 3.2 Habitat Manipulation: Experimental Treatment

A visualisation of behavioural variation per station across replicates is provided in Figure S2. For experimental treatment cleaning stations (*n* = 10), mean cleaning rates ± standard deviation per replicate were 4.65 ± 5.54, 5.03 ± 5.57, 3.59 ± 5.60 and 4.20 ± 5.53 cleans per hour for each respective replicate observations (Before, First, Second, After). Overall mean cleaning durations were 13.11 ± 21.23, 20.15 ± 21.28, 5.61 ± 21.36 and 22.69 ± 21.35, respectively. Mean posing rates were 7.34 ± 6.01, 5.00 ± 6.03, 2.94 ± 6.05 and 6.10 ± 6.07 poses per hour, respectively. Mean cleaner responsiveness for each replicate was 10.75 ± 2.27, 13.54 ± 3.69, 14.51 ± 3.67 and 13.9 ± 2.72, respectively.

Model output summaries are provided in Table S1. All ICC values were above 0.05, indicating that variation in Station ID and station surface area contributed meaningfully to the models, with total ICC varying from 0.10 to 0.41 (Table S2).

**FIGURE 1:**
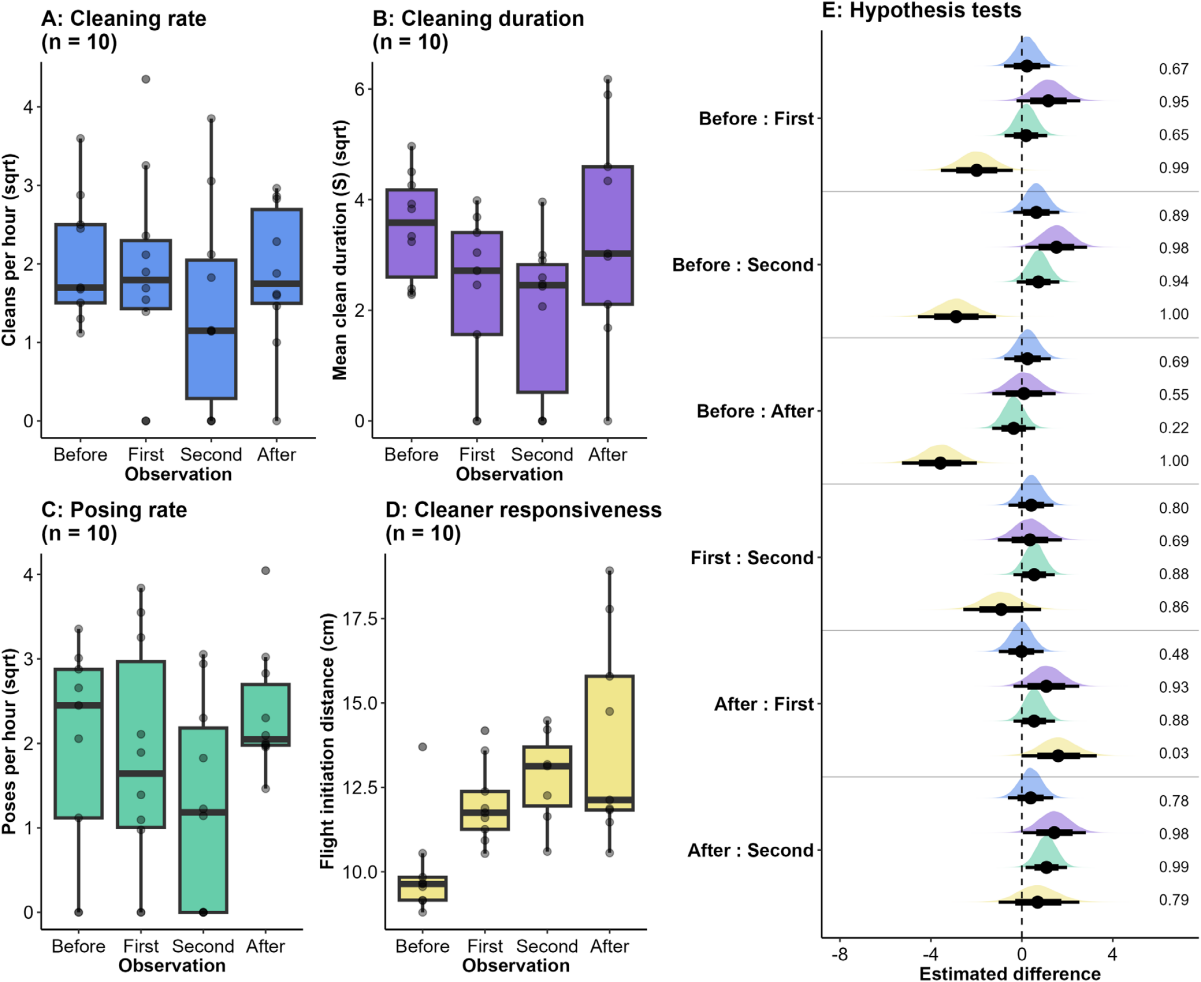
Variation in four mutualism‐associated behaviours before, during and after cleaning station habitat manipulation using a repeated measures design (*n* = 10). A–D: Raw data for each mutualism‐associated behaviour across the duration of the study. Points are individual cleaning stations. Boxplot lines show median values, and box length indicates interquartile ranges. Whiskers are the 25th and 75th percentiles. E: Density plots showing the extent to which behaviours (represented by consistent colours) vary across the four replicate observations for each behaviour. Points are medians, and 75% and 95% CIs are shown by thick and thin lines, respectively. Densities to the right of 0 indicate behaviours were higher in the observation indicated on the left of the y‐axis labels compared to the label on the right of the y‐axis labels, whilst densities to the left of 0 indicate behaviours were lower in the observation indicated on the left of the y‐axis labels. Numbers to the right of the density plots are posterior probabilities (PPs) that show the strength of the direction of each density plot. For cleaning rate, cleaning duration and posing rate, density plots (E) are based on square‐root transformed values.

For the cleaning rate, there was evidence of a decline between the baseline and second manipulation observations (Estimate: 0.51 (−0.21, 1.48), PP: 0.89, ER: 8.31, Figure 1A,E) and between the first and second manipulation observations (Estimate: 0.50 (−0.42, 1.23), PP: 0.80, ER: 3.93, Figure 1A,E). For cleaning duration, there was strong evidence of a decline between the baseline and first manipulation observation (Estimate: 1.17 (0.00, 2.33), PP: 0.95, ER: 18.81, Figure 1B,E) and the baseline and second manipulation observation (Estimate: 1.52 (0.39, 2.65), PP: 0.98, ER: 63.52, Figure 1B,E). For the posing rate, there was strong evidence of a decline between the baseline and second manipulation observation (Estimate: 0.48 (−0.06, 1.50), PP: 0.94, ER: 14.48, Figure 1C,E) and between the first and second manipulation observations (Estimate: 0.46 (−0.22, 1.30), PP: 0.88, ER: 7.52, Figure 1C,E). There was little evidence of a difference between the baseline observations and the final observations following the manipulation for the cleaning rate (Estimate: 0.52 (−0.60, 1.09), PP: 0.69, ER: 2.21, Figure 1A,E), cleaning duration (Estimate: 0.09 (−1.08, 1.25), PP: 0.55, ER: 1.23, Figure 1B,E) and posing rate (0.48 (−1.14, 0.42), PP: 0.22, ER: 0.28, Figure 1C,E).

There was a clear increase in cleaner FID between the baseline and first manipulation observation (Estimate: −1.98 (−3.30, −0.67), PP: 0.99, ER: 112.31, Figure 1D,E), between the baseline and the second manipulation observation (Estimate: 0.48 (−0.06, 1.50), PP: 1.00, ER: 739.74, Figure 1D,E) and a moderate increase between the first and second manipulation observations (Estimate: −0.90 (−2.30, 0.55), PP: 0.86, ER: 5.99, Figure 1E). There was also no evidence that cleaner FID returned to pre manipulation levels, as cleaner FID remained higher during the final observation following the manipulation compared to the baseline observation (Estimate: −3.59 (−4.97, −2.26), PP: 1.00, ER: 7999.00, Figure 1E).

**FIGURE 2:**
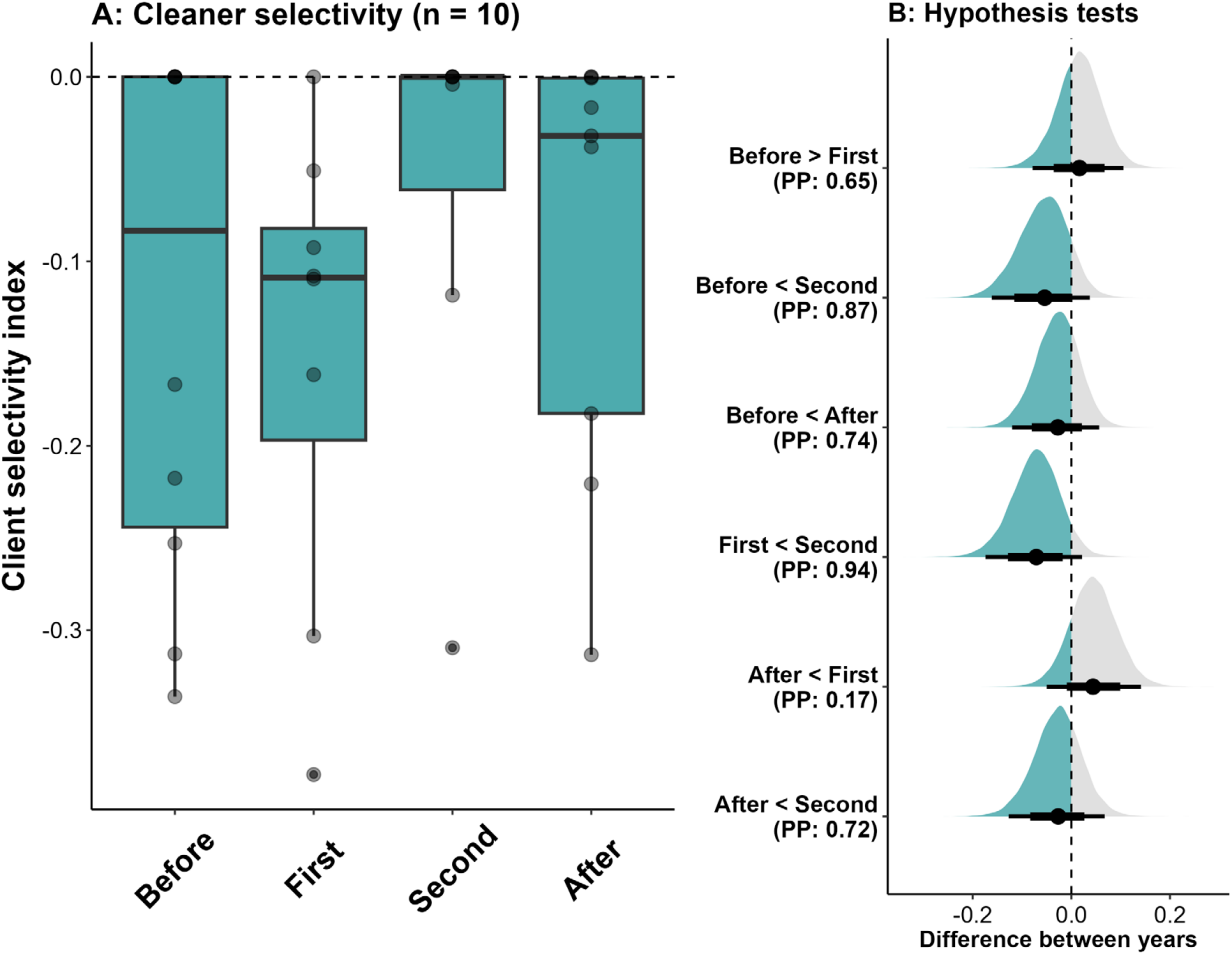
Variation in cleaner selectivity across the duration of the habitat manipulation experiment. A: Raw selectivity values for each replicate observation. Points are individual cleaning stations. B: Density plots showing the extent to which cleaner selectivity showed an increase (Green) or a decrease (grey) across the replicate observations. Points are medians, and thick and thin lines are 75% and 95% Cis, respectively. Y‐ axis labels indicate the direction and the strength (measured as the posterior probability, PP) of each tested hypothesis.

Mean cleaner selectivity was −0.13 ± 0.14, −0.11 ± 0.12, −0.10 ± 0.13 and −0.11 ± 0.11 for each respective replicate observation. There was some evidence that cleaner selectivity was higher at the second manipulation observations compared to the baselines (Estimate: −0.06 (−0.14, 0.02), PP: 0.87, ER: 6.98, Figure 2, Table S1) and higher at the first manipulation observation (Estimate: −0.07 (−0.16, 0.01), PP: 0.94, ER: 14.53, Figure 2, Table S1). There was little evidence that cleaner selectivity was variable before and after manipulations (Estimate: −0.03 (−0.10, 0.04), PP: 0.74, ER: 2.82, Figure 2, Table S1).

**FIGURE 3:**
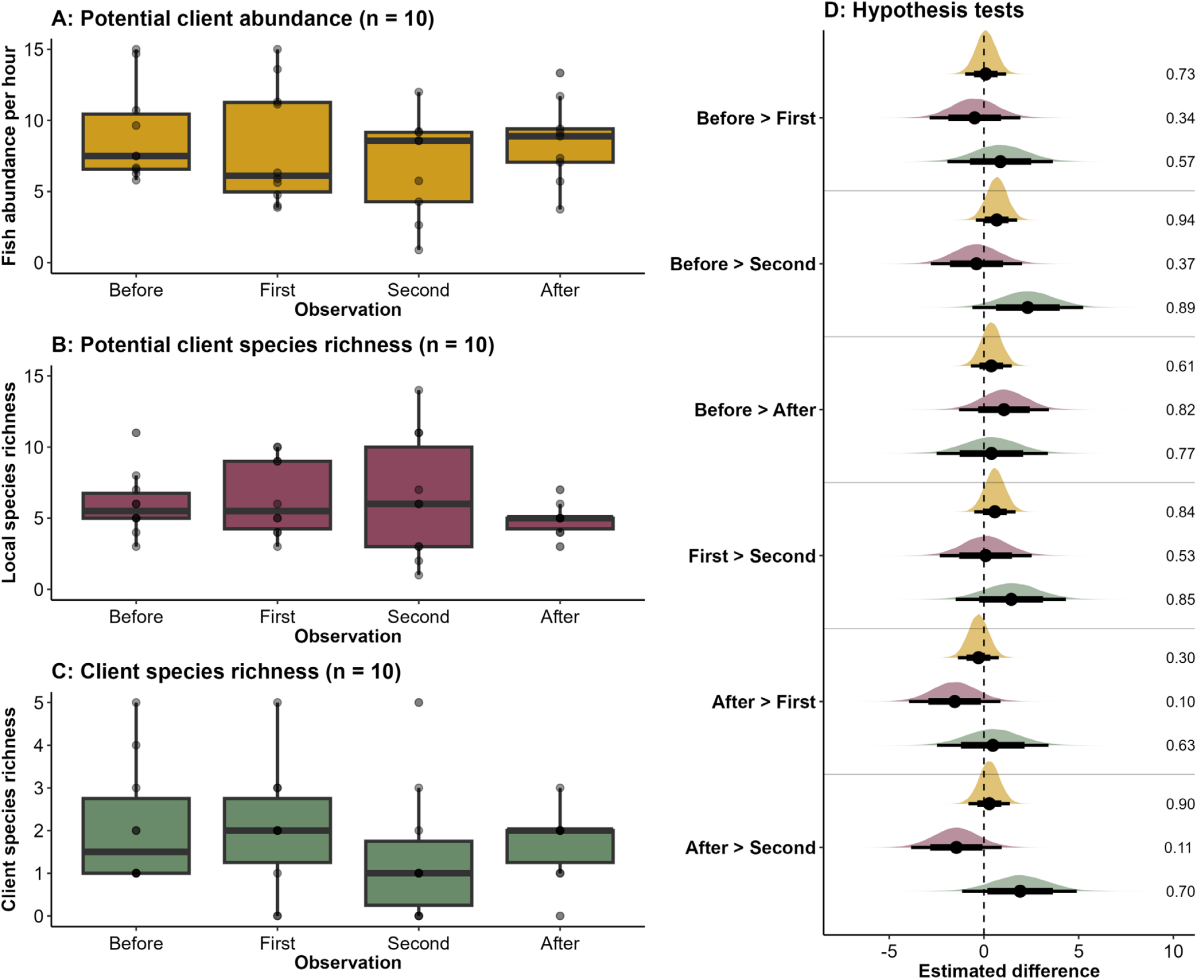
Variation in the local reef fish and client community across the habitat manipulation experiment. A–C: Raw data for each variable. Points represent an individual cleaning station. D: Density plots show the extent to which the response variables (represented by consistent colours) vary across different replicate observations based on hypothesis tests. Points are medians, and thick and thin lines are 75% and 95% Cis, respectively. The direction and strength (PP) for each hypothesis are shown in the Y‐axis labels.

Whilst overall, the declines apparent for cleaner–client behaviours above are not as apparent for the abundance and richness of the local reef fish community (Figure 3A), there was some evidence that client species richness declined between the baseline observation and the second manipulation observation (Estimate: 0.67 (−0.24, 1.58), PP: 0.89, ER: 7.98, Figure 3B, Table S1). There was also little evidence that following the manipulations, the client species richness was lower than the baseline observations (Estimate: 0.39 (−0.51, 1.29), PP: 0.77, ER: 3.27, Figure 3B, Table S1).

### 3.3 Habitat Manipulation: Differences Relative to Controls

For the models comparing the absolute differences in response variables for experimental cleaning stations relative to those of control stations, all ICC values were above 0.10; total ICC varied between 0.29 and 0.69 (Table S2).

**FIGURE 4:**
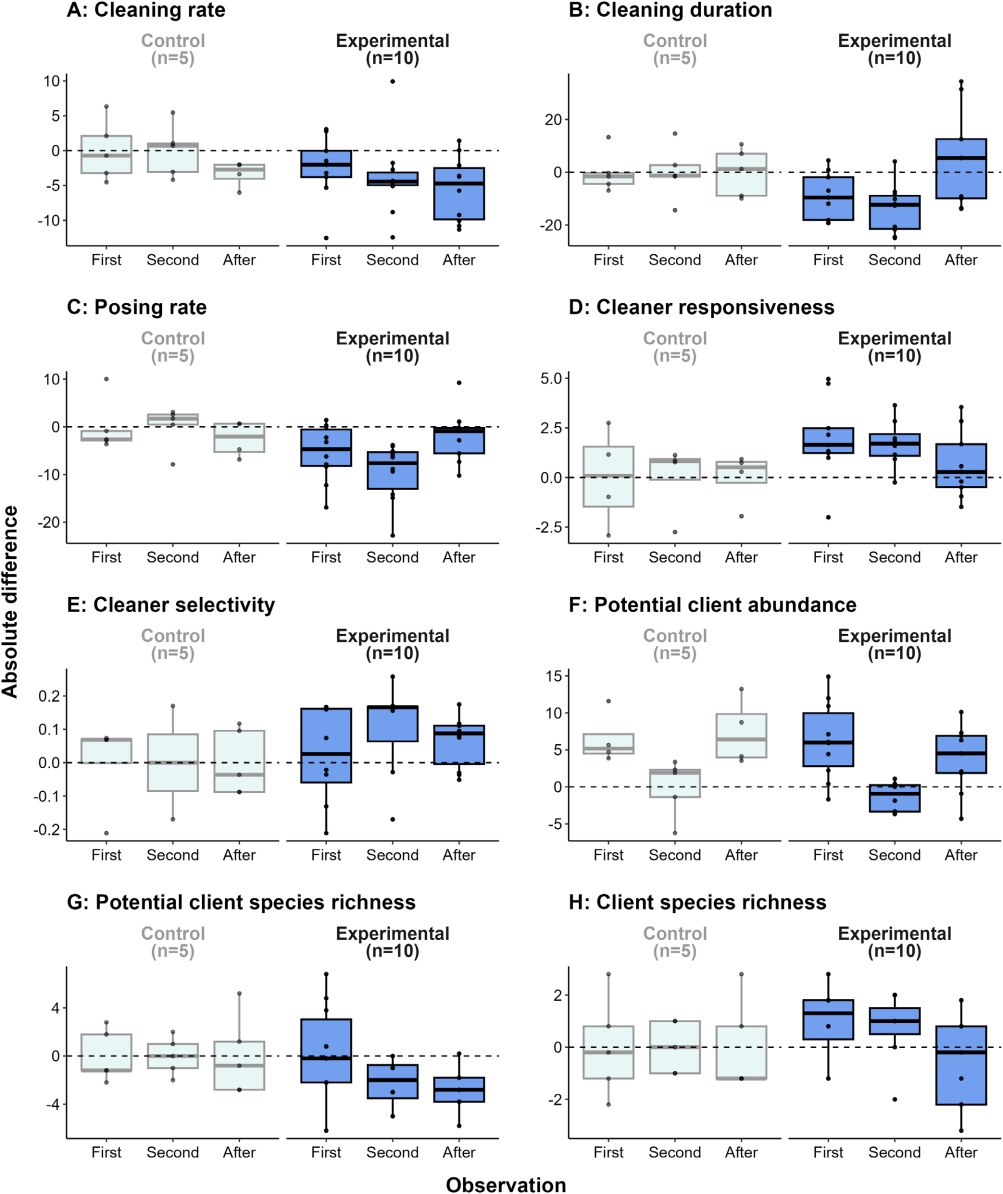
Absolute differences in the eight response variables across the duration of the habitat manipulation experiment for experimental stations relative to control cleaning stations. Points are individual cleaning stations. For the control stations, absolute differences for each observation are based on the difference between the current observation and the previous observation. For experimental stations, absolute differences for any given observation are based on the mean rate of change value for the equivalent observation at control stations.

For cleaning rate, cleaning duration, posing rate and cleaner responsiveness, there was generally strong evidence for a negative absolute difference in behaviours between the baseline and first manipulation observations (Cleaning rate PP: 0.84, cleaning duration PP: 0.91, posing rate PP: 0.95, cleaner responsiveness PP: 0.95, Figure 4A–D, Table 1) and between the first and second manipulation observations (Cleaning rate PP: 0.93, cleaning duration PP: 0.98, posing rate PP: 0.95, cleaner responsiveness PP: 0.95, Figure 4A–D, Table 1) relative to the control stations. Additionally, there was little evidence that the absolute differences for experimental stations were different from those of control stations between the second manipulation observations and observations after the manipulation, for cleaning rate (PP: 0.77, Figure 4A, Table 1), cleaning duration (PP: 0.73, Figure 4B, Table 1), posing rate (PP: 0.53, Figure 4C, Table 1) and cleaner responsiveness (PP: 0.70, Figure 4D, Table 1).

**TABLE 1:** Summary of model estimates and hypothesis tests for the absolute differences of eight response variables at experimental cleaning stations compared to controls.

	Observation	Hypothesis	Estimate (± 95% CI)	Evidence ratio	Posterior probability
Cleaning rate	First	C>E	2.67 (−1.79, 5.25)	5.25	0.84
	Second	C>E	3.87 (−0.56, 8.25)	12.37	0.93
	After	C>E	2.06 (−2.50, 6.66)	3.44	0.77
Cleaning duration	First	C>E	9.34 (−2.08, 20.89)	10.19	0.91
	Second	C>E	13.72 (2.41, 25.04)	40.15	0.98
	After	C<E	−4.28 (−15.68, 7.23)	2.74	0.73
Posing rate	First	C>E	5.53 (0.01, 11.06)	19.07	0.95
	Second	C>E	9.53 (4.01, 15.11)	267.46	1.00
	After	C>E	0.29 (−5.55, 6.24)	1.13	0.53
Cleaner responsiveness	First	C<E	−1.91 (−3.85, 0.04)	17.94	0.95
	Second	C<E	−1.61 (−3.60, 0.41)	9.91	0.91
	After	C<E	−0.63 (−2.58, 1.33)	2.37	0.70
Cleaner selectivity	First	C<E	0.00 (−0.14, 0.13)	1.09	0.52
	Second	C<E	−0.07 (−0.23, 0.08)	3.71	0.79
	After	C<E	−0.06 (−0.19, 0.06)	3.93	0.80
Potential client abundance	First	C<E	−0.52 (−5.02, 3.91)	1.35	0.58
	Second	C>E	0.86 (−3.51, 5.19)	1.71	0.63
	After	C>E	2.37 (−2.27, 6.89)	4.19	0.81
Potential client species richness	First	C>E	−0.31 (−2.95, 2.34)	0.73	0.42
	Second	C>E	2.06 (−0.70, 4.79)	8.35	0.89
	After	C>E	2.69 (−0.05, 5.44)	18.00	0.95
Client species richness	First	C<E	−1.01 (−2.74, 0.59)	6.05	0.86
	Second	C<E	−0.88 (−2.58, 0.80)	4.17	0.81
	After	C>E	0.53 (−1.08, 2.15)	2.47	0.71

Absolute differences in cleaner selectivity for experimental cleaning stations were not variable to the rate of change predicted at control stations between the baseline and first manipulation observations (PP: 0.52, Figure 4E, Table 1). There was also little evidence of variation in the absolute differences between experimental and control stations between the first and second manipulation observations (PP: 0.79, Figure 4E, Table 1).

In general, there was little variation in absolute differences of potential client abundance during the duration of the study relative to control stations (Figure 4F, Table 1). There was evidence of a negative absolute difference between both the first and second manipulation observations (PP: 0.89, Figure 4G, Table 1) and second manipulation observations and observations following manipulation (PP: 0.95, Figure 4G, Table 1) at experimental stations relative to controls for potential client species richness. For client species richness, there was evidence of a positive absolute difference at experimental stations relative to controls for both between the baseline and first manipulation observations (PP: 0.86, Figure 4H, Table 1) and between the first and second manipulation observations (PP: 0.81, Figure 4H, Table 1).

Finally, there was little variation in the absolute differences for all response variables across the duration of the study for experimental controls compared to controls (Figure S3, Table S3), with the exception of cleaner responsiveness. Cleaner responsiveness was higher at the first replicate observation compared to controls (PP: 0.99, Figure S3, Table S3).

## 4 Discussion

Marine cleaning mutualisms promote biodiversity, and obligate cleaners are keystone species in structuring marine mutualistic networks and subsequently coral reef ecosystem function (Quimbayo et al. 2018). We provide evidence of a consistent decline in the quality and quantity of cleaning interactions in response to a short‐term habitat disturbance. Reduced access to live habitat resulted in changes in the expression of cleaner (cleaning rate, duration, selectivity, and responsiveness) and client (posing rate) behaviours, beyond expected natural rates of change over time. Mutualism‐associated behaviours also returned to pre‐disturbance levels after the manipulation was removed. Marine cleaning mutualisms may therefore show a level of resilience via behavioural plasticity in response to short‐term environmental disturbance.

A loss of specialisation between mutualistic parties has been documented in terrestrial (Ferreira et al. 2020) and marine (Triki et al. 2018) systems in response to environmental stressors. For obligate cleaner species that rely directly on a mutualism for nutritional resources (Baliga and Mehta 2019), a change in foraging strategy, e.g., a decline in service quality (duration), should be supplemented by an increase in service quantity, i.e., rate (Gunn and Michiels 2025). However, we identified consistent declines in cleaning rate, duration and cleaner selectivity, a proxy for mutualistic specialisation, over a period of just 11 days, even though we reduced habitat, not nutritional (client) resource availability. In fact, potential client availability remained unaffected throughout the experiment. Changes in one aspect of a mutualism can therefore have direct and indirect cascading effects for all interacting parties.

In the context of our experiment, cleaner FID perhaps reflects cleaner ‘boldness’ (Réale et al. 2007) as a response to a risky scenario, with the increased responsiveness (higher FID) reflecting a lower level of boldness as cleaners ‘flee’ from the object sooner. This could also explain the unexpected variation in cleaner FID between control and experimental control stations with open nets. Boldness can predict foraging behaviours across different taxonomic groups (Pereira et al. 2020) and has been linked to the strategies via which individuals balance food and fear (Mella et al. 2015). Reduced access to refuges/live habitat could increase the perceived risk to cleaners of engaging in mutualistic behaviours. Cleaners at cleaning stations with a higher availability of refuges also display higher cleaning rates and duration (Whittey et al. 2021). An increase in perceived risk due to reduced habitat access could explain the decrease in boldness as a fear response, which in turn resulted in suppressed foraging behaviours. Nonetheless, whilst cleaning behaviours reverted to pre‐disturbance levels after the manipulation, cleaner boldness (FID) did not.

Cleaning stations likely provide a refuge effect against predation (Cheney et al. 2008) and cleaners have a chemical defence against predators (Tuttle et al. 2021). Despite this, *Elacatinus* cleaners still show increased stress (cortisol) levels toward predatory clients, but this does not suppress cleaning activity (Soares et al. 2012). High levels of stress hormones (e.g., cortisol) are linked to reduced boldness in fishes (Raoult et al. 2012; Alfonso et al. 2019) and terrestrial vertebrates (Huang et al. 2024). The reversibility of behaviours that directly maintain the mutualism on which cleaners rely for nutrients may therefore occur faster than indirect behaviours such as boldness that may also have a physiological component. The maintenance of reduced boldness related to foraging during periods of unpredictability is also predicted to increase fitness by avoiding the costs associated with high‐risk environments (Luttbeg and Sih 2010). Habitat loss may therefore disrupt behavioural nuances associated with high‐quality cleaner–client interactions via indirectly affecting the costs:benefit ratio of engaging in a cleaning interaction.

Habitat loss is a key driver of declines in mutualist abundance and mutualistic services (Winfree et al. 2011). There is a mismatch between the length of the study (days) and the time over which environmental change would reduce habitat availability (weeks‐months). Our manipulation caused an immediate loss of habitat that perhaps induced more exacerbated responses than would be seen on a real‐world disturbance timescale. However, immediate behavioural responses are expected where animals are directly affected, i.e., the cleaners, whereas a longer time lag in responses may be more likely where animals are indirectly affected by a disturbance, i.e., the client community (Rahman and Candolin 2022). This was true of our system, such that cleaner‐associated behaviours generally showed greater changes than client‐associated metrics.

Whilst the benefits of marine cleaning mutualisms take years to become ecologically relevant (Clague et al. 2011), habitat loss can impact cleaning station quality within weeks (Alvarez‐Filip et al. 2011). Multiple aspects of the cleaning mutualism (cleaning rate and duration, posing rate and client species richness) did show evidence of plasticity back to pre‐disturbance levels. Behavioural plasticity is favoured in fluctuating environments as a mechanism to promote species persistence (Ducatez et al. 2020). Short‐term, in situ manipulations allow the initial responses and plasticity of species interactions to be quantified under natural conditions. However, the ’Before’ baseline does not necessarily directly reflect the true natural baseline of an undisturbed system due to the ongoing bleaching event (NOAA Coral Reef Watch 2024), even though focal cleaning stations were healthy. We suggest future work uses longer‐term in situ experiments across reefs of different habitat qualities. This would eliminate temporal mismatches between experimental manipulations and real disturbances and allow responses from different baselines of disturbance to be quantified. Combined, short‐ and long‐term experiments can then be used to predict the long‐term persistence of species interactions. Given the extent of the declines in cleaner and client behaviours quantified in this study, the observed behavioural plasticity, we suggest that there will be a point beyond which behavioural plasticity is no longer a viable response strategy, resulting in a mutualism breakdown (Kiers et al. 2010).

Both the true (*N* = 20) and effective (station level replication) sample sizes of the study are relatively small. ‘Moderate’ posterior probabilities should therefore not be overinterpreted. For example, reef fish communities did not show substantial variation across the study period. But we cannot decouple whether this is a result of resilience from the reef fish community against reduced habitat availability, or simply a product of low statistical power. Nonetheless, despite the small sample sizes and the well‐documented context‐dependency of mutualistic interactions (Chamberlain et al. 2014), we were still able to document consistent behavioural changes, with high posterior probabilities, throughout the habitat manipulation.

We have given a novel, local‐scale insight into how marine cleaning mutualisms respond to short‐term habitat disturbances. We suggest that, in the short term, cleaners display behavioural plasticity and reversibility in response to such disturbances. Predicting whether positive species interactions break down under environmental change or function as a buffer against such change is vital for susceptible ecosystems with complex interaction networks such as coral reefs (Lewis and Altieri 2025). Finally, expanding upon short‐term in situ manipulations to identify tipping points and thresholds beyond which keystone species interactions such as marine cleaning mutualisms collapse is an important future research goal for predicting and preserving the persistence of one of the world's most threatened ecosystems.

## Author Contributions

**R. L. Gunn:** conceptualization (lead), data curation (lead), formal analysis (lead), funding acquisition (lead), investigation (lead), methodology (lead), project administration (lead), resources (lead), validation (lead), writing – original draft (lead), writing – review and editing (equal). **C. G. Obst:** data curation (supporting), methodology (supporting), writing – review and editing (equal). **P. Vetter:** data curation (supporting), methodology (supporting), writing – review and editing (equal).

## Funding

This work was supported by the RiSC funding programme from the Ministeriums für Wissenschaft und Kunst Baden‐Württemberg (PRO‐GUNN‐2023‐06).

## Disclosure

Statement on inclusion. Our work does bring together authors from different countries, albeit from the same institution. Our work unfortunately fails to include scientists from the country in which the work was carried out. We acknowledge and recognize that our work is therefore missing the diverse perspectives that the inclusion of local authors and stakeholders could provide. Whilst Operation Wallacea engages with local communities and institutions, there is a scarcity of local scientists working in our study area. We acknowledge this caveat and aim to address this issue in future research by engaging local students in research via an internship programme.

## Conflicts of Interest

The authors declare no conflicts of interest.

## Supporting information


**Figure S1:** Summary of the variation in cleaning station surface area (A) and cleaning station rugosity (B) between treatments, and the association between cleaning station surface area and cleaning station rugosity (C). A and B: Points represent individual cleaning stations. The control treatment consists of combined data for control stations and experimental control stations. Therefore, *n* = 10 for both treatments. C: Posterior distribution of the predicted negative correlation between cleaning station surface area and cleaning station rugosity, as predicted from a Bayes Factor (BF) test. The PP represents the likelihood that a negative correlation is present.
**Figure S2:** Line plots to visualise behaviours at each individual experimental cleaning station across replicates for experimental cleaning stations (*N* = 10). Each coloured line represents an individual cleaning station, with colouring consistent across panels A–G. Cleaning stations for which data was missing from any replicate observation has been removed.
**Figure S3:** Absolute differences in the eight response variables across the duration of the habitat manipulation experiment for experimental control stations relative to control cleaning stations. Points are individual cleaning stations. For the control stations, absolute differences for each observation are based on the difference between the current observation and the previous observation. For experimental control stations, absolute differences for any given observation are based on the mean rate of change value for the equivalent observation at control stations.
**Table S1:** Summary of models and hypothesis tests for eight response variables for experimental cleaning stations across the duration of the habitat manipulation experiment.
**Table S2:** Summary of all Bayesian models, including intraclass correlation coefficients (ICCs) for random effects, R‐hat values, bulk effective sample sizes (ESS) and Bayesian R^2^ estimates.
**Table S3:** Summary of model estimates and hypothesis tests for absolute differences of eight response variables at experimental control cleaning stations relative to controls.

## Data Availability

Data and code for this work will be made available via Figshare (10.6084/m9.figshare.31486009) and GitHub (https://github.com/rlgunn1/Cleaning_mutualism_habitat_manipulation).
