# Supplementary material for: Response and Plasticity of a Cleaning Mutualism Following Short Term Reductions in Habitat Availability
Source: Ecol Evol. 2026 Apr 16;16(4):e73348. doi: 10.1002/ece3.73348 (PMC13085738; doi:10.1002/ece3.73348)
Supplement: Supplementary file 1 — Figure S1: Summary of the variation in cleaning station surface area (A) and cleaning station rugosity (B) between treatments, and the association between cleaning station surface area and cleaning station rugosity (C). A and B: Points represent individual cleaning stations. The control treatment consists of combined data for control stations and experimental control stations. Therefore, n = 10 for both treatments. C: Posterior distribution of the predicted negative correlation between cleaning station surface area and cleaning station rugosity, as predicted from a Bayes Factor (BF) test. The PP represents the likelihood that a negative correlation is present. Figure S2: Line plots to visualise behaviours at each individual experimental cleaning station across replicates for experimental cleaning stations (N = 10). Each coloured line represents an individual cleaning station, with colouring consistent across panels A–G. Cleaning stations for which data was missing from any replicate observation has been removed. Figure S3: Absolute differences in the eight response variables across the duration of the habitat manipulation experiment for experimental control stations relative to control cleaning stations. Points are individual cleaning stations. For the control stations, absolute differences for each observation are based on the difference between the current observation and the previous observation. For experimental control stations, absolute differences for any given observation are based on the mean rate of change value for the equivalent observation at control stations. Table S1: Summary of models and hypothesis tests for eight response variables for experimental cleaning stations across the duration of the habitat manipulation experiment. Table S2: Summary of all Bayesian models, including intraclass correlation coefficients (ICCs) for random effects, R‐hat values, bulk effective sample sizes (ESS) and Bayesian R2 estimates. Table S3: Summary of model estimates [file ECE3-16-e73348-s001.docx]

**Supplementary methods**

Cleaner selectivity

The availability of clients was quantified such that:

$$p=\frac{\left( \frac{t_{i}I_{i}}{S_{i}} \right)}{\sum_{n=0}^{n=i} \left( \frac{t_{i}I_{i}}{S_{i}} \right)}$$

Where *t* is the number of time intervals (screengrabs) in which each client species was present, *I* is the abundance of each client species and *S* is the total number of screengrabs taken from each cleaning station.

**Supplementary results**


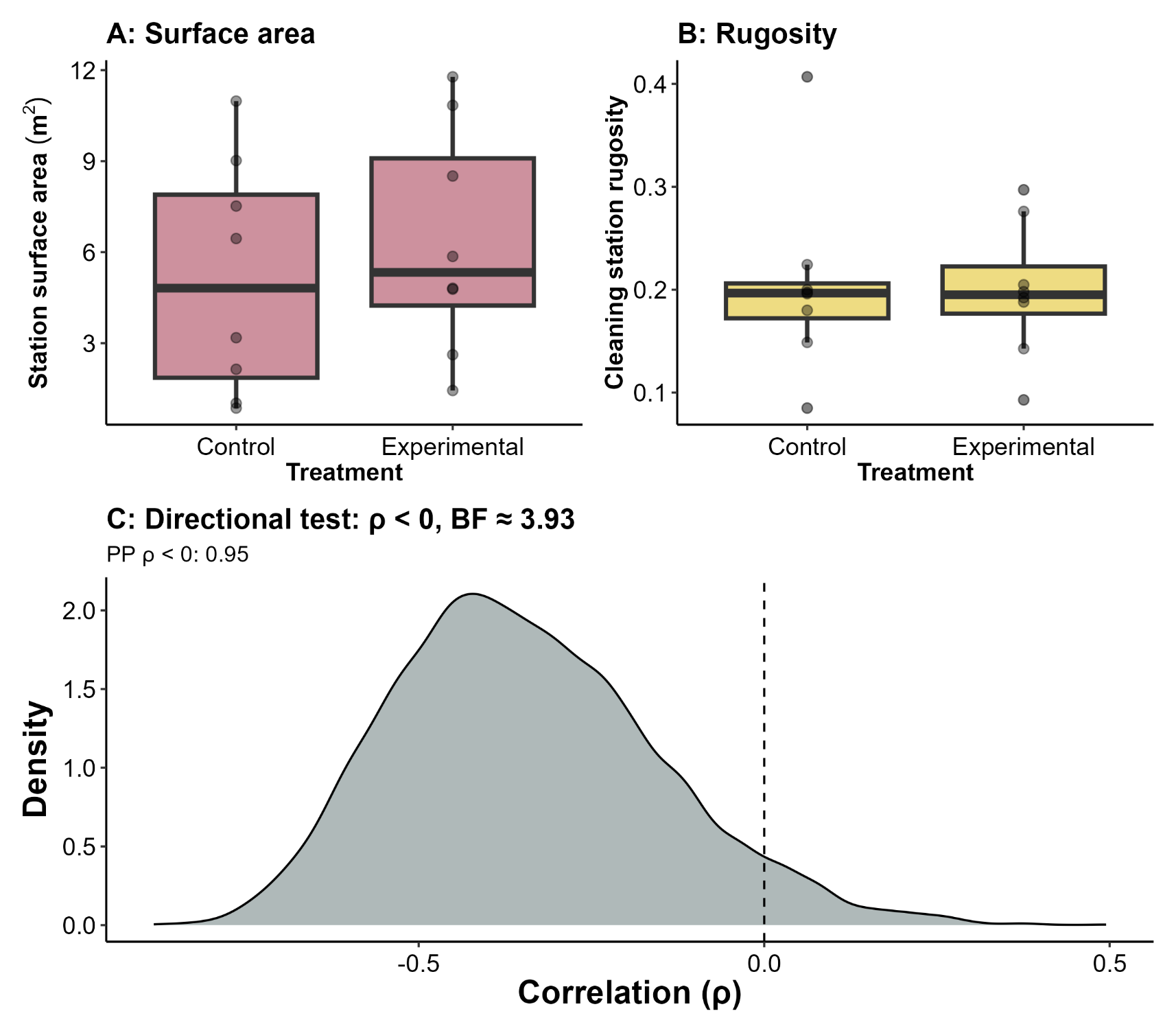
**Figure S1: Summary of the variation in cleaning station surface area (A) and cleaning station rugosity (B) between treatments, and the association between cleaning station surface area and cleaning station rugosity (C).** A&B: Points represent individual cleaning stations. The control treatment consists of combined data for control stations and experimental control stations. Therefore, n=10 for both treatments. C: Posterior distribution of the predicted negative correlation between cleaning station surface area and cleaning station rugosity, as predicted from a Bayes Factor (BF) test. The PP represents the likelihood that a negative correlation is present.

**
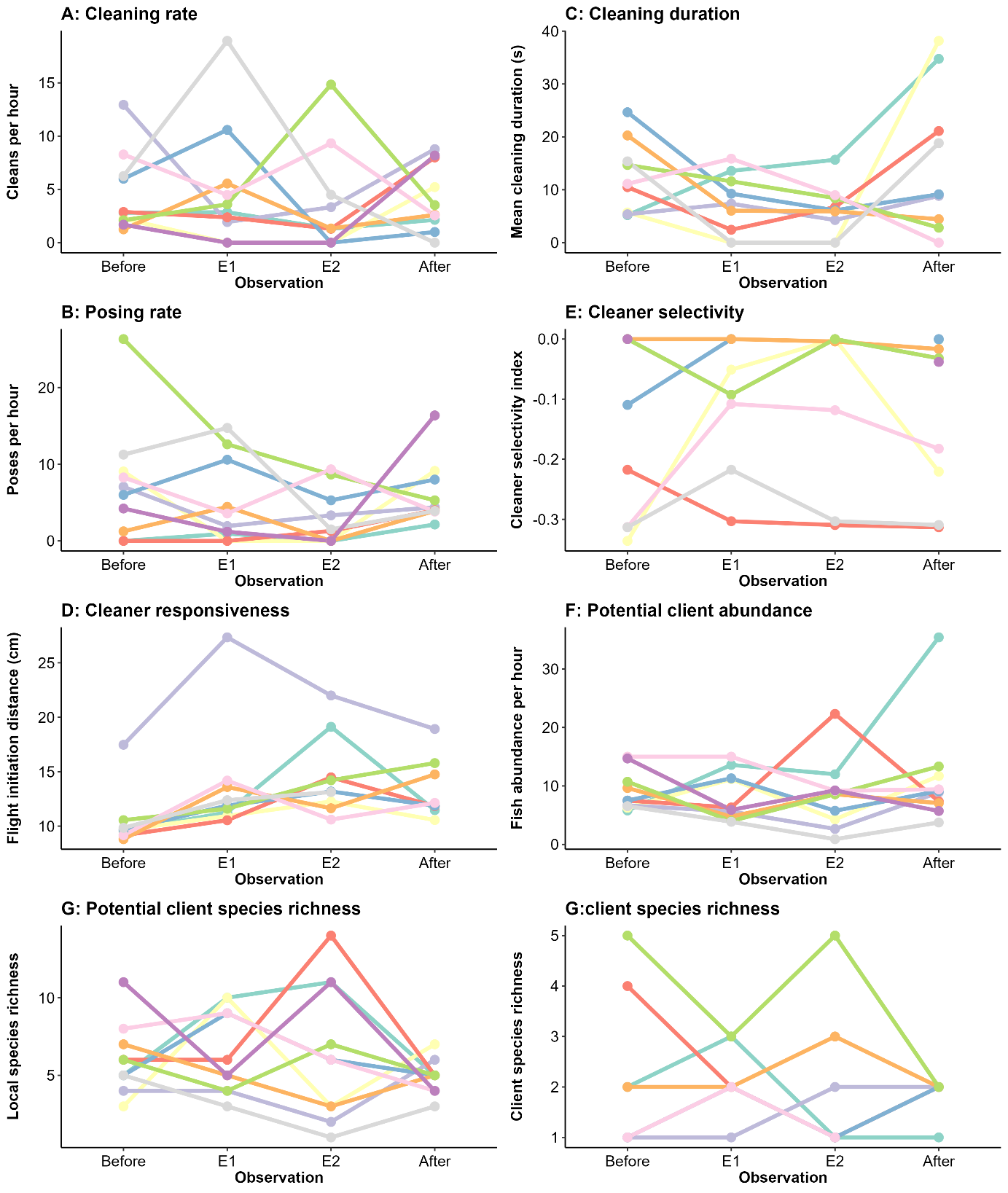
**

**Figure S2: Line plots to visualise behaviours at each individual experimental cleaning station across replicates for experimental cleaning stations (N=10).** Each coloured line represents an individual cleaning station, with colouring consistent across panels A-G. Cleaning stations for which data was missing from any replicate observation has been removed.

**Table S1: Summary of models and hypothesis tests for eight response variables for experimental cleaning stations across the duration of the habitat manipulation experiment.**

|  | **Hypothesis** | **Estimate**  **(± 95% CI)** | **Evidence ratio** | **Posterior probability** |
| --- | --- | --- | --- | --- |
| **Cleaning rate** | Before > First | 0.51 (-0.62, 1.06) | 2.07 | 0.67 |
|  | Before > Second | 0.51 (-0.21, 1.48) | 8.31 | 0.89 |
|  | Before > After | 0.52 (-0.60, 1.09) | 2.21 | 0.69 |
|  | First > Second | 0.50 (-0.42, 1.23) | 3.93 | 0.80 |
|  | After > First | -0.02 (-0.85, 0.80) | 0.94 | 0.48 |
|  | After > Second | 0.50 (-0.44, 1.21) | 3.58 | 0.78 |
| **Cleaning duration** | Before > First | 1.17 (0.00, 2.33) | 18.81 | 0.95 |
|  | Before > Second | 1.52 (0.39, 2.65) | 63.52 | 0.98 |
|  | Before > After | 0.09 (-1.08, 1.25) | 1.23 | 0.55 |
|  | First > Second | 0.35 (-0.81, 1.52) | 2.25 | 0.69 |
|  | After > First | 1.08 (0.27, 2.59) | 13.46 | 0.93 |
|  | After > Second | 1.43 (0.27, 2.59) | 44.82 | 0.98 |
| **Posing rate** | Before > First | 0.47 (-0.60, 0.96) | 1.87 | 0.65 |
|  | Before > Second | 0.48 (-0.06, 1.50) | 14.48 | 0.94 |
|  | Before > After | 0.48 (-1.14, 0.42) | 0.28 | 0.22 |
|  | First > Second | 0.46 (-0.22, 1.30) | 7.52 | 0.88 |
|  | After > First | 0.54 (-0.21, 1.30) | 7.54 | 0.88 |
|  | After > Second | 0.46 (0.32, 1.84) | 87.11 | 0.99 |
| **Cleaner responsiveness** | Before < First | -1.98 (-3.30, -0.67) | 112.31 | 0.99 |
|  | Before < Second | -2.88 (-4.28, -1.45) | 739.74 | 1.00 |
|  | Before < After | -3.59 (-4.97, -2.26) | 7999.00 | 1.00 |
|  | First < Second | -0.90 (-2.30, 0.55) | 5.99 | 0.86 |
|  | After < First | 1.61 (0.25, 3.01) | 0.03 | 0.03 |
|  | After > Second | 0.71 (-0.74, 2.22) | 3.75 | 0.79 |
| **Cleaner selectivity** | Before < First | 0.02 (-0.06, 0.09) | 1.82 | 0.65 |
|  | Before < Second | -0.06 (-0.14, 0.02) | 6.98 | 0.87 |
|  | Before < After | -0.03 (-0.10, 0.04) | 2.82 | 0.74 |
|  | First < Second | -0.07 (-0.16, 0.01) | 14.53 | 0.94 |
|  | After > First | 0.04 (-0.03, 0.12) | 0.20 | 0.17 |
|  | After > Second | -0.03 (-0.11, 0.05) | 2.56 | 0.72 |
| **Potential client abundance** | Before > First | -0.48 (-2.47, 1.52) | 0.52 | 0.34 |
|  | Before > Second | -0.40 (-2.40, 1.61) | 0.59 | 0.37 |
|  | Before > After | 1.06 (-0.91, 3.02) | 4.41 | 0.82 |
|  | First > Second | 0.09 (-1.93, 2.10) | 1.13 | 0.53 |
|  | After > First | 0.47 (-1.96, 2.90) | 1.69 | 0.63 |
|  | After > Second | -1.46 (-3.46, 0.54) | 0.13 | 0.11 |
| **Potential client species richness** | Before > First | 0.88 (-1.45, 3.21) | 2.75 | 0.73 |
|  | Before > Second | 2.31 (-0.11, 4.73) | 16.34 | 0.94 |
|  | Before > After | 0.41 (-2.00, 2.85) | 1.56 | 0.61 |
|  | First > Second | 1.44 (-1.00, 3.86) | 5.14 | 0.84 |
|  | After > First | -1.54 (-3.55, 0.47) | 0.11 | 0.10 |
|  | After > Second | 1.91 (-0.61, 4.39) | 8.64 | 0.90 |
| **Client species richness** | Before > First | 0.10 (-0.80, 0.99) | 1.34 | 0.57 |
|  | Before > Second | 0.67 (-0.24, 1.58) | 7.98 | 0.89 |
|  | Before > After | 0.39 (-0.51, 1.29) | 3.27 | 0.77 |
|  | First > Second | 0.58 (-0.33, 1.48) | 5.80 | 0.85 |
|  | After > First | -0.29 (-1.19,0.61) | 0.42 | 0.30 |
|  | After > Second | 0.28 (-0.63, 1.20) | 2.32 | 0.70 |

**Table S2: Summary of all Bayesian models, including Intraclass correlation coefficients (ICCs) for random effects, R-hat values, bulk effective sample sizes (ESS) and Bayesian R^2^ estimates.**

|  | **Variable** | **Random effects ICC** | | | **Bulk ESS** | **Rhat** | **Bayes R^2^** |
| --- | --- | --- | --- | --- | --- | --- | --- |
|  |  | **Station ID** | **Station surface area** | **Total** |  |  |  |
| **Experimental data** | Cleaning rate | 0.06 | 0.06 | 0.12 | 48578 | 1.00 | 0.19 |
|  | Cleaning duration | 0.05 | 0.05 | 0.10 | 35037 | 1.00 | 0.26 |
|  | Posing rate | 0.15 | 0.15 | 0.30 | 36053 | 1.00 | 0.36 |
|  | Cleaner responsiveness | 0.20 | 0.20 | 0.41 | 19189 | 1.00 | 0.56 |
|  | Cleaner selectivity | 0.19 | 0.20 | 0.38 | 22394 | 1.00 | 0.38 |
|  | Potential client abundance | 0.14 | 0.13 | 0.27 | 25756 | 1.00 | 0.32 |
|  | Potential client species richness | 0.09 | 0.09 | 0.17 | 38667 | 1.00 | 0.24 |
|  | Client species richness | 0.14 | 0.14 | 0.28 | 28167 | 1.00 | 0.31 |
| **Absolute difference**  **(Control vs Experimental)** | Cleaning rate | 0.15 | 0.15 | 0.30 | 27934 | 1.00 | 0.44 |
|  | Cleaning duration | 0.16 | 0.17 | 0.33 | 28305 | 1.00 | 0.52 |
|  | Posing rate | 0.22 | 0.23 | 0.45 | 23462 | 1.00 | 0.60 |
|  | Cleaner responsiveness | 0.06 | 0.06 | 0.13 | 33351 | 1.00 | 0.33 |
|  | Cleaner selectivity | 0.35 | 0.34 | 0.69 | 19761 | 1.00 | 0.72 |
|  | Potential client abundance | 0.22 | 0.22 | 0.44 | 22212 | 1.00 | 0.63 |
|  | Potential client species richness | 0.15 | 0.15 | 0.29 | 28053 | 1.00 | 0.45 |
|  | Client species richness | 0.17 | 0.17 | 0.34 | 23457 | 1.00 | 0.43 |
| **Absolute differences**  **(Control vs Experimental control)** | Cleaning rate | 0.19 | 0.19 | 0.38 | 24536 | 1.00 | 0.44 |
|  | Cleaning duration | 0.19 | 0.19 | 0.38 | 31371 | 1.00 | 0.39 |
|  | Posing rate | 0.19 | 0.19 | 0.38 | 27044 | 1.00 | 0.37 |
|  | Cleaner responsiveness | 0.10 | 0.10 | 0.21 | 32300 | 1.00 | 0.51 |
|  | Cleaner selectivity | 0.11 | 0.11 | 0.21 | 30167 | 1.00 | 0.36 |
|  | Potential client abundance | 0.07 | 0.07 | 0.14 | 36563 | 1.00 | 0.33 |
|  | Potential client species richness | 0.26 | 0.26 | 0.52 | 20407 | 1.00 | 0.51 |
|  | Client species richness | 0.19 | 0.19 | 0.39 | 20230 | 1.00 | 0.46 |


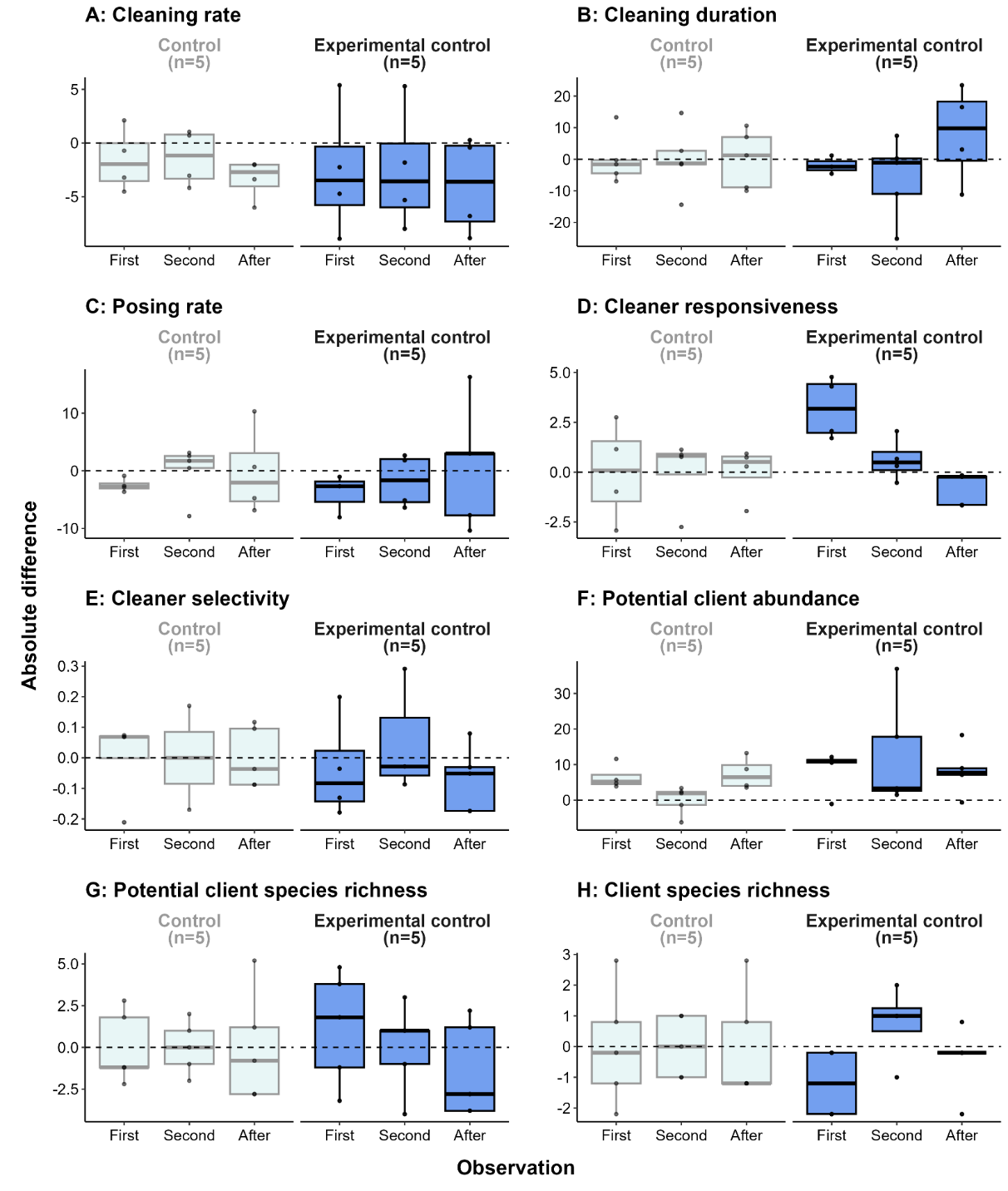


**Figure S3: Absolute differences in the eight response variables across the duration of habitat manipulation experiment for experimental control stations relative to control cleaning stations.** Points are individual cleaning stations. For the control stations, absolute differences for each observation are based on the difference between the current observation and the previous observation. For experimental control stations, absolute differences for any given observation are based on the mean rate of change value for the equivalent observation at control stations.

**Table S3: Summary of model estimates and hypothesis tests for absolute differences of eight response variables at experimental control cleaning stations relative to controls.**

|  | **Replicate** | **Hypothesis** | **Estimate**  **(± 95% CI)** | **Evidence ratio** | **Posterior probability** |
| --- | --- | --- | --- | --- | --- |
| **Cleaning rate** | First | C > EC | 1.88 (-3.49, 7.25) | 2.65 | 0.73 |
|  | Second | C > EC | 1.92 (-3.42, 7.30) | 2.72 | 0.73 |
|  | After | C > EC | -0.12 (-5.46, 5.24) | 0.94 | 0.49 |
| **Cleaning duration** | First | C > EC | 2.99 (-11.39, 17.27) | 1.77 | 0.64 |
|  | Second | C > EC | 5.80 (-7.03, 18.57) | 3.50 | 0.78 |
|  | After | C > EC | -7.82 (-21.07, 5.45) | 0.19 | 0.16 |
| **Posing rate** | First | C > EC | 2.82 (-6.04, 11.70) | 2.39 | 0.71 |
|  | Second | C > EC | 2.24 (-5.64, 10.13) | 2.16 | 0.68 |
|  | After | C > EC | -0.77 (-8.70, 7.11) | 0.77 | 0.43 |
| **Cleaner responsiveness** | First | C < EC | -3.22 (-5.45, -1.00) | 87.50 | 0.99 |
|  | Second | C < EC | -0.64 (-2.89, 1.61) | 2.22 | 0.69 |
|  | After | C < EC | 0.79 (-1.36, 2.93) | 0.36 | 0.27 |
| **Cleaner selectivity** | First | C > EC | 0.03 (-0.17, 0.24) | 1.52 | 0.60 |
|  | Second | C > EC | -0.06 (-0.30, 0.19) | 0.53 | 0.35 |
|  | After | C > EC | 0.07 (-0.12, 0.25) | 2.62 | 0.72 |
| **Potential client abundance** | First | C > EC | -1.98 (-10.76, 6.88) | 0.54 | 0.35 |
|  | Second | C > EC | -10.69 (-19.05, -2.02) | 0.02 | 0.02 |
|  | After | C > EC | -0.76 (-9.54, 8.08) | 0.79 | 0.44 |
| **Potential client species richness** | First | C > EC | -1.18 (-4.39, 2.04) | 0.37 | 0.27 |
|  | Second | C > EC | -0.01 (-3.29, 3.23) | 1.00 | 0.50 |
|  | After | C > EC | 1.36 (-1.88, 4.61) | 3.16 | 0.76 |
| **Client species richness** | First | C > EC | 1.16 (-0.72, 3.02) | 5.72 | 0.85 |
|  | Second | C > EC | -0.90 (-2.78, 0.97) | 0.26 | 0.21 |
|  | After | C > EC | 0.39 (-1.39, 2.18) | 1.82 | 0.65 |
